# Individual play patterns stimulated by a familiar object are group-driven

**DOI:** 10.1038/s41598-019-42382-9

**Published:** 2019-04-15

**Authors:** Manja Zupan, Therese Rehn, Daiana de Oliveira, Špela Malovrh, Linda Keeling

**Affiliations:** 1 Swedish University of Agricultural Sciences, Department of Animal Environment and Health, P. O. Box 7068, 750 07 Uppsala, Sweden; 2 University of Ljubljana, Biotechnical Faculty, Department of Animal Science, Groblje 3, 1230 Domžale, Slovenia

## Abstract

This study investigates the dynamics of play behaviour within groups of four juvenile pigs and uses a novel clustering and statistical modelling approach to describe new details in how individuals play with a familiar object (toy rope). We examined complex state sequence data collected during a 30 min home pen play test, using the package TraMineR, where the states were defined as object play, locomotor/social play and no play. From behavioural observations, and based on the relative proportion of the different types of object play observed, each individual was later categorised as an initiator or joiner type of player. Initiators were found to be more solitary and to show more object play whereas joiners were more social and showed less object play. The majority of groups did not have an initiator type of player, yet on average they played more. Despite strong group and type of player effects, we identified three general individual play patterns. On a group level, our results demonstrate differences in how a period of playing develops, that playing with the object simultaneously occurs more often in groups than expected by chance and that the number of pigs playing together is stable over time.

## Introduction

Animal welfare scientists agree that play is an important indicator of good animal welfare^1^ and that promoting it may be a useful tool to improve housing conditions for captive animals such as chimpanzees^2^ or commercial animals such as domestic pigs^3^. Animals usually play when their primary needs are met, i.e., they are healthy, satiated and in the absence of negative stressors or threat^4^. However, in species like primates, laboratory and companion animals as well as humans^2,5^, play increase has been observed also in situations with threats to fitness or in aversive situations. Considering neurobiological evidence, play is likely to be a subjectively pleasurable activity for animals, as for humans^6,7^. Play can be categorized into four different play types; social, object, predatory or locomotor play^8^, each consisting of specific elements of play behaviour. When playing, animals show great variability in play patterns, as indicated by different sequences in play behaviour elements^9^. If certain play patterns are combined and expressed in a consistent way, animals can be classified into different groups according to play styles^10^. For better understanding of the different play terms used in this manuscript, we summarized them in the Supplementary Table S1 and provided their descriptions.

From a social evolutionary perspective, play is important in the development and the maintenance of social ties, enabling individuals to interact with each other for the benefits of all group members, and also to develop and maintain communication skills^9,11^. Proposing the social cohesion hypothesis of play^12^, playing together was argued also to increase cohesion within the social group, which can be evident in species like the spotted hyena (*Crocuta crocuta*, ref.^13^), but not in other species like the meerkats (*Suricata suricatta*, ref.^14^). Further it was emphasized that the complexity of social relationships was a crucial driver in the development of the primate brain^15^ and that play contributes to the development of skills necessary to navigate social relationships as adults^16^. This and other increasing evidence^17–19^ for the adaptive benefits of play illustrate the function of social play, which is to promote complex socio-cognitive development (e.g., play-mediated learning) and behavioural flexibility. More specifically, social play functions to strengthen affiliative ties between group members, although a recent example in primates^2^ suggests it can only reduce social tension and not necessarily indicate that individuals form affiliative social relationships.

Even under commercial husbandry conditions, animals like fattening pigs show a variety of complex social interactions, where individuals assess social relationships and show emotional contagion^20^, a simple form of empathy^21^. Pigs thus provide a powerful mammalian species model within which to gain information about decision-making strategies encountered during affiliative situations. There has been work on the expression of different play types, and the factors influencing them, in pigs kept in groups^22–27^ and in pairs^28^. Nevertheless, there are many details of how animals in groups play that are not yet described. Improved understanding of these details could contribute to our understanding of the evolutionary significance of mammalian play and, perhaps, open up new avenues of how to stimulate play behaviour in commercial pigs.

In this study, our primary interest was to gain new knowledge of group dynamics during play, by investigating how often individuals initiate play behaviour and the variation in the sequences of juveniles play within a single 30 min play session. We chose to build groups of four pigs (*Sus scrofa domesticus*) since Conradt^29^ argued that a group of four has characteristics of a group up to ten, which is close to the group size in commercial pig farming practice. Also, the social structure of four pigs is more complex than in pairs, which poses new challenges, and is more likely to represent a natural situation^30^. We hypothesized that not only companion^9^ or laboratory animals^31^, but also commercial animals express different individual play patterns. We also hypothesized that individual play patterns are affected by group dynamics.

## Results

To investigate the group play dynamics, 15 groups, each consisting of four sibling pigs, were exposed to a 30 min play test in their home pen with two familiar play objects (tug toy ropes; 21305 records). A general linear model (GLM) showed no significant effect of body weight, sex nor previous handling differences (P > 0.05) in any of the sequence analyses.

### Play behaviour

All pigs performed all play types (for their descriptions see Table 1). Pigs approached a toy within 16.6 ± 22.7 s (mean ± SD) from the start of the test (ranging from 5 s to 115 s). During the test, pigs spent more than half of their time playing (56.6%) with most time being allocated to performing object play (ObjP; 49.2%; on the ground 39.0% and off the ground 10.2%). Playing alone with a toy on the ground was found to be positively correlated with playing with a toy off the ground (r = 0.73; P < 0.0001). Pigs performed locomotor play (LocP; 5.1%) and social play (SocP; 2.3%) at low frequencies.

**Table 1:** Behaviour scored in the home pen play test.

Behaviour	Play type	Description		Elements
Play	Social play (SocP)	Play directed at conspecifics		Nosing, mouting, lever, biting, head knocks, nosing, following/chasing
	Locomotor play (LocP)	Movement of the body		Pivot, turn, scamper, hop, flop, head toss
	Object play (ObjP)	Play directed at a toy	Initiating	The animal starts to interact with a toy first
			Joining	The animal joins to interact with a toy while another pig is already interacting with it
			Alone	The animal plays with a toy on the ground or off the ground alone
			Together	The animal plays with a toy on the ground in groups of 2, 3 or 4; object play off the ground with the same toy could not be performed in groups
	No play (NoP)	Activities not related to play		—
Exploration		Latency to approach a toy		—

Based on the relative proportion of time an individual was seen initiating or joining object play, it was later classified as an initiator, a joiner or a mixed ‘type of player’ in the analysis. Similarly, depending on the relative proportion of time an individual was seen playing with the object alone or together with one or more other pigs, it was later classified as a solitary, social or mixed ‘type of player’ for the analysis.

Based on number of times an individual was observed in the four sub-descriptions of object play, mentioned in Table 1, we were able to reveal the inter-individual variation of play behaviour in individual pigs within this 30 min play session. We identified different types of players. Pigs were classified as initiators (I; observed mainly initiating object play), joiners (J; observed mainly joining a pig who was already playing with the object) or mixed type of player (I/J; if it could not be classified). Similarly, pigs were defined as solitary (observed mainly playing with the object alone) or social (observed mainly playing with the object together with one or more other pigs), otherwise pigs were set as a mixed type of player (if it could not be classified). There was a significant overlap of these two types of player classifications (Kendall’s rank correlation tau = −0.614. ASE = 0.069; P < 0.05; Table 2). Noteworthy, initiators were all found to be solitary whereas joiners were social or mixed players (solitary/social) and social individuals were joiners or mixed players (initiators/joiners). Thus, although similar, the solitary/social classification is not a perfect overlap with the initiator/joiner classification. A pig who initiated play with an object played ‘alone’ until either it stopped playing with the object or it was joined by another pig. But if it was joined by another pig and both continued to play, then the initiator was now playing ‘together’. Thus it cannot be assumed that initiators are solitary players. Similarly, a pig who joined a pig already playing with the object was playing ‘together’, but if the first pig stopped playing, the joiner pig was now playing ‘alone’. Thus neither can it be assumed that joiners are social players. Initiators and joiners did not differ in sex (Fisher’s Exact test; Chi-square = 5.35; P > 0.05; data not shown) nor in birth weight or weight at 9 weeks (Kruskal-Wallis Test; Chi-square = 4.09; P > 0.05; data not shown).

**Table 2:** A contingency table on the number of animals in the initiator/joiner or solitary/social classification.

Frequency	Solitary	Mixed (solitary/social)	Social
Initiator	6	0	0
Mixed (initiator/joiner)	9	14	3
Joiner	0	14	14

**Figure 1:**
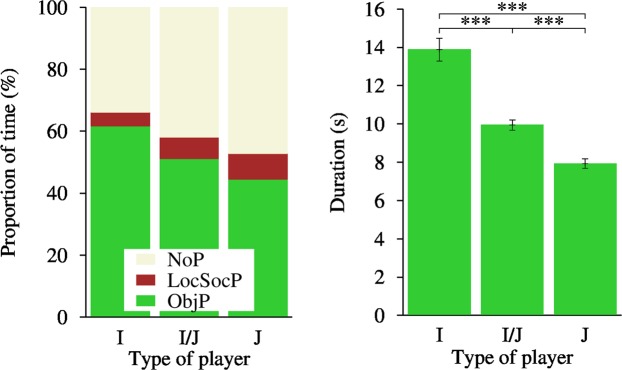
The allocation of time spent on different play types (left; calculated on absolute frequencies of states) and the duration of sequences of ObjP (right) by type of player during 30 min in a home pen play test. NoP-no play activities; ObjP-object play; LocSocP-locomotor and social play; I-initiator; J-joiner; I/J-mixed type of player; ***P < 0.0001.

Investigating the association between play behaviour and ‘type of player’, initiators performed less LocSocP and more ObjP compared to joiners and mixed players (Fig. 1 left; df = 4, Chi-square = 30.68, P ≤ 0.0001). Initiators approached a toy quickest and joiners the latest (I vs. J: t-value = −7.82, P < 0.0001; J vs. I/J: t-value = −8.77, P < 0.0001; J vs. I/J: t-value = 2.81, P = 0.007).

Groups of pigs spent different proportions of the total test time playing (ranging from 30.5% to 72.1%; Supplementary Table S2; Mantel-Haenszel Chi-square test based on absolute frequencies of states; Chi-square = 52.6, P < 0.0001) but the distribution of the different types of players within these groups did not differ significantly (Supplementary Table S2; Fisher Exact test, P = 0.976). When comparing groups with or without initiators, we found that groups without initiators spent significantly higher proportions of time in object play (Chi-square = 24.5, P < 0.0001) as well as in locomotor/social play (Chi-square = 48.8, P < 0.0001).

### Sequences of play

In the initial analysis eight play states were identified. We refer to these states as ‘play types’, which is a common term for the more statistically correct term ‘play states’. They were: no play (NoP), object play (ObjP), locomotor play (LocP), social play (SocP), and when they occurred simultaneously (object + locomotor play, object + social play, locomotor + social play, and object + locomotor + social play). Following preliminary analyses, LocP, SocP and their combinations were found to occur infrequently and so were merged (LocSocP). Similarly, the occurrences of play with the object on and off the ground, as well as all the possible combinations with object play were also combined, but still called ObjP. These meant that three play types were included in the final analysis; no play, object play and a combined locomotor and social play.

We found that the sequence of ‘play types’ was significantly different between groups of pigs and between types of players (Supplementary Table S3) and also that these differences were apparent within the three measures of the sequence complexity; turbulence, complexity index and longitudinal entropy (Supplementary Table S4). Similarly, when looking at the durations of the sequences of ObjP, groups of pigs had significantly different durations (df = 14, F-value = 4.56; P < 0.0001) and initiator pigs had longer sequences of ObjP than joiner pigs (Fig. 1 right; df = 2, F-value = 46.51; P < 0.0001). As the 30 min progressed, the sequences of ObjP got shorter (estimate = −0.00025 ± 0.00; df = 1, F-value = 252.36; P < 0.0001).

### Transitions between play types

We computed the probability of transitions between play types. We found that the NoP and ObjP play types were very stable (no change in behaviour in around 80% of successive 5 s observations) but that this was not the case for LocSocP (in bold, Supplementary Table S5). It is shown that LocSocP followed NoP or ObjP on very low frequencies suggesting that LocSocP was likely not to be influenced by the play stimulus. This is further supported by LocSocP following equally frequent all three play types and not exclusively ObjP. After performing LocSocP, the pig was as likely to stop playing (NoP) as it was to still be performing LocSocP. It was always the case that the initiator type of player pigs made fewer transitions than joiners (Supplementary Table S5; Cochran-Mantel-Haenszel test; P-value < 0.0001). Initiators made less frequent changes from NoP to ObjP (difference = −0.0485), from NoP to LocSocP (difference = −0.0272), ObjP to NoP (difference = −0.0927) and from ObjP to LocSocP (difference = −0.0305). As a consequence, in addition to the longest sequences of ObjP, initiator pigs were also more consistent in performing ObjP and NoP (ObjP → ObjP difference I − J = 12.32%; NoP → NoP difference I − J = 7.57%).

### Identifying individual play patterns

**Figure 2:**
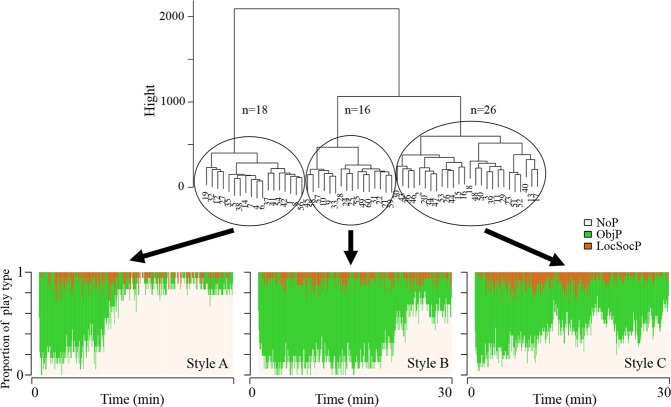
Cluster dendrogram (above) and the three clusters of pigs depending on the sequence of play (below). NoP-no play activities; ObjP-object play; LocSocP-locomotor and social play.

Beside the strong group and type of player effects (Supplementary Table S4) on the sequences of play, three clusters of pigs (A, B and C) who showed different overall play patterns were found in the cluster analysis (Fig. 2). The difference between the three clusters was in the proportion of ObjP and for how long animals kept playing with a toy (Mantel-Haenszel Chi-square test; Chi-Square = 58.81; P < 0.0001). Most individuals were allocated to play pattern C. They performed more ObjP than individuals with play pattern A, who stopped playing with a toy after around 10 min, but less than individuals in pattern B. LocSocP was performed consistently throughout the observations in all the clusters, but most often in pigs with pattern C.

### Development of play during test

**Figure 3:**
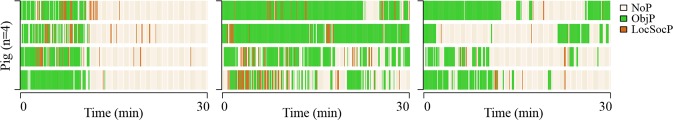
Sequence index plots: Examples of the state sequences performed during 30 min in a home pen play test in groups of four pigs. Y-axis: each row represents an individual pig. Pigs from the 1^st^ group were mainly playing in the first 10 min while pigs from the 2^nd^ group were playing during the whole time with some short periods of no play. Pigs from the 3^rd^ group showed an inconsistency in how they played.

Looking at the group level, differences in the development of play over the 30 min were observed. Some groups were playing very little, others played almost all the time and others played intermittently. This diversity is illustrated by the three graphs in Fig. 3. We also found that pigs from the same group were significantly more likely to be individuals from the same play pattern cluster (Fisher-Exact test; P = 0.0085) but there was no relationship with the proportion of initiators and joiners in the group. This again demonstrates that there is a difference between the type of player.

### Synchronisation of play

To see how synchronised pigs are during play we looked at the number of pigs playing simultaneously. Although the degree of synchrony in play differed between groups of pigs (df = 28, Chi-square = 376.3, p-value < 0.0001), no specific pattern could be found. Social play directed at conspecifics by definition must involve at least two individuals, but that three or even all four pigs were playing simultaneously within a 5 s interval was found significantly more often than expected both for social play (95% CI, 16.7–23.2%) and locomotor play (95% CI, 41.2–46.8%; both P < 0.05). Since LocP and SocP occurred at very low frequencies, in contrast to ObjP, we could not compute the transition rates of the number of individual pigs playing simultaneously for this play type, not even for LocSocP.

Pigs were playing simultaneously with a toy, although not necessarily with the same toy, mostly in pairs (27.9%; Supplementary Table S6) and least in groups of four (12.4%). Pigs used both toys instead of sharing the same toy for 60.9% of the time when playing with the toy in pairs, 72.0% when playing in trios and 85.4% of the time when playing in tetrads. The three different characteristics of ObjP are shown in Supplementary Table S6. Firstly, stability of number of pigs involved in ObjP was found, as indicated on the diagonal by the fact that the same number of individuals played together for longer than 5 s in almost 50% of cases. Secondly, joining another pig (or pigs) already playing occurred about one fifth of the time (from 1 pig to 2 pigs 19.6%; from 2 to 3 pigs 22.9%; from 3 to 4 pigs 16.5%), which is significantly higher than the probability of two pigs playing together by chance (ca. 7%), and much higher than the probability of three (ca. 2%) or four pigs playing together (ca. 0.5%). Thirdly, playing in a group was a gradual process, not an ‘all start’ or ‘all stop’ process. It was rare that if no pig was playing (preceding no. of pigs 0) in the next interval all four pigs were playing (following no. of pigs 4; 0.1%). Similarly, if three or four pigs were playing, it was unlikely that in the next interval no pig was playing (less than 0.2%).

## Discussion

The main finding of this study is that when given the opportunity to play with toys in a familiar environment for 30 min, play behaviour in pigs was group-driven despite the fact that each individual animal has its own specific pattern of play during this time. Although we cannot confirm that the patterns we observed are individual behavioural strategies stable over a longer time or whether an individual uses a strategy on an ad hoc basis, our results support the hypothesis that individual play patterns can develop in a mammalian farm animal. We used a statistical approach for mining and visualizing sequences of categorical data known in social sciences for sequence analysis^32^, but which to our knowledge has never been applied in animal behaviour studies previously.

The play behaviours were categorised as play pattern A, B and C (Fig. 2). Such clustering, which depended on different quantities of play behaviour and variation in play types, may suggest that on an individual level, animals develop different strategies in an object play context. Play patterns as different play styles have been proposed previously in other mammals such as rats^31^ and dogs^10^. In these studies animals were observed in different situations and occasions indicating individual behavioural strategies that were repeatable and consistent. In contrast to these studies, we observed the animals in a group-context rather than in pairs, but we did it only on one single observation period. Almost half of our pigs adopted the strategy of a relatively consistent level of object play during the 30 min of test (pattern C) and of performing more locomotor and social play compared to pigs with play patterns A and B.

Regardless of the allocation to a particular play pattern, an individual was classified as the specific type of player, based on the proportion of its involvement in object play as a pig who initiated play with an object or it was joined by another pig. This phenotypic characteristic was found to be closely associated with the sociality traits of an individual, with initiators being individualists whereas joiners were more social players. This means even though there were the individuals that took the lead in initiating object play, they did not keep on playing when another pig joined. Initiators by definition most often reached the play object the quickest, however not due to sex or body weight. Such characteristics may, according to models of motivation^33^, indicate that some individuals within a group have a higher motivation to explore an object/environment or a lower fear or anxiety level^34^. If this is true, and we consider the interaction with an object, particularly on the ground, as a measure of exploration with its basic function to inform an individual about the object^35^, then it is not surprising that our initiators played with the object most, but performed the least locomotor and social play (Fig. 1 left). They also had the longest durations of sequences of object play (Fig. 1 right) and made the least behavioural changes (Supplementary Table S5). Their play behaviour could be viewed as a strategy to have a low energy cost expenditure^36^, so enhancing early survival, and is supported by the study in free-living ground squirrels (*Spermophilus beldingi*; ref.^37^) suggesting nonsocial play to be less energetically costly than social play.

Only a small proportion of the pigs in this study were classified as initiators and the majority of groups did not have an initiator (Supplementary Table S2). The groups without initiators were observed to be more playful (i.e., showing more object play and more locomotor/social play). That the majority of pigs were classified as social or the mixed solitary/social type of player may imply that acting social in the play context is more important than acting solitary. The difference between groups with and without an initiator suggests different social organisation possibilities in a play context. For example, having a pig motivated for solitary object play may somehow limit the play behaviour of the other pigs and lead to a different group play dynamics. Different types of play are suggested to have different types of beneficial effects from an evolutionary functional perspective^9^. Object play helps to develop skills concerned with physical problem solving, social play to aid in establishing social hierarchies and to tie social bonds^15^ whereas locomotor-rotational play helping to practice dexterity and motor skills^38^.

Despite a strong inter-individual variability in play behaviour, our results suggest that individual play patterns are group determined. When looking at the transitions in the state sequences (i.e., changes from one type of play to another), we found that groups of pigs had a significantly different development of play during the 30 min test (Fig. 3) which was not under the influence of the relative balance of initiators or joiners. By showing that within the groups, the four pigs had mainly the same play pattern (i.e., were grouped into the same cluster), we argue, although speculative, that pigs developed a democratic group decision-making mechanism. This argumentation is based on the model of Conradt and Roper^39^, where the authors described the despotically communal decisions when one dominant decides to act (comparable in our study to the pig who initiates object play) and democratically communal decisions when there is a majority of group members deciding (comparable in our study to when several pigs initiated object play).

Showing a certain degree of behavioural synchronisation, likely also due to shared genetics and early experience, by sharing the same play object with two or more conspecifics (Supplementary Table S6), and performing locomotor and social play with more than two individuals at the same time, may further support the idea that juvenile play behaviour is group-driven. It may be fun to play with an object alone, especially if you are a solitary individualist, but it is more fun playing with it together with others. We found that playing simultaneously with the object occurred more often in groups than expected by chance and did not develop rapidly if there was nobody playing, nor did it stop rapidly if there were more individuals playing. Group size during object play was stable over time (Supplementary Table S6), however during the process of playing, there was dynamic in the number of animals playing due to pigs joining or leaving group play.

Play is considered to be behaviour with a motivational state of liking^6^ and finding strong group-determined play behaviour in pigs, supports that play is a pleasurable socio-emotional interaction. There is some documentation^40–43^ showing that decision-making processes of individuals are influenced by the presence of conspecifics, also whether or not an individual is playing^40^. Maintaining proximity and showing synchronised behaviour during exploration and play allows animals to continuously exchange information about their emotional states^40,44^. Such behaviour responses may enable an individual pig to emotionally benefit from being in a group^45^ or enabling the best fitness consequences by, for instance, improving individual abilities to gain food resources as well as increasing the chance of survival^9^.

Our work provides new evidence that individual play patterns stimulated by a familiar object are group-driven. This would be in keeping with the idea of adaptive play strategies and play styles but because we only observed the pigs on one occasion this still needs to be confirmed. What is also new about this study is the novel application of statistical methodologies to the behaviour data. We applied two methods to analyse complex state sequences, the cluster analysis and the statistical modelling of sequence data. This statistical approach allowed us to demonstrate that individual decisions of what play type to perform or for how long to play are strongly influenced by communal decisions.

## Materials and Methods

### Ethical statement

This study was approved by the Uppsala Ethical Committee of Animal Experimentation, Uppsala, Sweden, under protocol C117/11. We hereby confirm that the study was performed in accordance with the relevant guidelines and regulations.

#### Animals

The experiment was carried out in 2011 at the Swedish Livestock Research Centre, Lövsta, at the Swedish University of Agricultural Sciences. We used 60, 9-week old crossbred piglets (Yorkshire X Hampshire) from 13 litters. The piglets were housed in individual farrowing pens (3.84 m × 2.2 m) with partly slatted concrete floors and straw. Commercial pelleted dry diets and water were available *ad libitum* for the piglets.

Piglets from 4 litters had been exposed to 23 sessions of 2 min standardised gentle handling^45^ from five days of age until weaning (35 days old). In four other litters none of the piglets were handled, while in five litters half of the piglets in the litter were handled. After weaning, piglets were exposed to a battery of different tests; (1) open-field/human approach test at four weeks of age^46^, (2) play/exploratory test in a new environment at five weeks of age^28^, and (3) a play test in a home pen environment at nine weeks of age, which is presented in this paper.

#### Home pen play test

Fifteen groups of four pigs from the same litter, balanced for body weight, sex and treatment (handled or non-handled pigs) participated. Each group was tested in its home pen and the littermates who were not being tested were temporarily removed. The test started when two tug toy ropes, known to the pigs from an earlier play/exploration test^17^, were thrown into the middle of the pen and it lasted for 30 min. Animals were recorded continuously using two video cameras and behaviour was later scored according to the ethogram (Table 1) using the Mangold Interact version 0.5.2.147^47^. One observer coded latency to approach a toy, SocP and ObjP. Two observers each coded LocP for half of the groups with 89% inter-observer agreement. The order of the video coding was randomised and the observers were blind to the handling treatments of the pigs. In addition to recording latency to approach the toy, instantaneous observations every 5 s were used (for ObjP, NoP), and one-zero recording within each 5 s (for LocP, SocP) to determine frequencies of interacting with the toy or another pig.

#### Statistical analysis

The statistical analysis was performed with the SAS package, version 9.4^48^ and with the R package, version 3.2.2^49^. In the text, only significant results are presented.

Sequences were prepared in a state sequence format. Each piglet had a sequence of 360 (30 min divided into 5 s intervals). Although LocP and SocP are sometimes of so short duration they could be seen as events, we treated them as states in order to get comparable data records to ObjP and NoP. To determine the degree of dissimilarity, the pair-wise optimal matching distances^50^ as implemented in TraMineR were computed among individual sequences. Insertion/deletion costs were set to 1 and observed transition rates matrix was used for substitution cost matrix. Cluster analysis was used to classify individual pigs’ sequences into a few different types of sequences. Agglomerative hierarchical clustering on obtained distance matrix was performed using Ward’s method^51^. The number of clusters was determined by average silhouette width^52^ and Hubert’s C index^53^.

After videos were coded off and the number of times an individual was observed in the four sub-descriptions of object play analyzed, it was classified a “type of player”. This was initiator, joiner or mixed initiator/joiner and solitary, social and mixed solitary/social type of player based on proportion of its involvement in object play with tug toy ropes as initiator or joiner and solitary or social. The one sample test of proportions based on 90% confidence intervals per pig was used to denote initiators and joiners. If proportion of initiating play was significantly higher than 0.5 that piglet was named a initiator and when that proportion was significantly lower than 0.5 that animal was named joiner. If proportion was not significant (p > 0.05) animal was denoted as mixed type. A similar procedure was performed to classify animals as solitary, social or mixed type player based on the proportion of their involvement in solitary and social object play.

Variation in individual sequence characteristics was analysed by within-sequence entropy, turbulence and complexity index^30^. These were computed using TraMineR for each pig and later analysed as traits by general linear model (GLM) where group (n = 15), treatment (n = 2; handled animals, non-handled animals), sex (n = 2; male, female), type of player (separately as n = 3 initiator, joiner, mixed type and n = 3; solitary, social, mixed type) were treated as fixed class effect, while birth weight and weight at 9 weeks were treated as covariates. Duration of sub-sequences of ObjP (i.e., number of successive occurrences) was computed for each pig and analysed by GLIMMIX using Poisson distribution with the same variables as above in the model, together with time as an additional covariate.

Transition rates between successive pairs of states were used to measure the probability of switching from one state to another at a given time (position) in a sequence. The states used were NoP, ObjP and LocSocP and transition rate matrices were computed for all 60 pigs, as well as for groups, sex and type of player. Differences in transition rates were assessed by a series of post-hoc Cochran-Mantel-Haenszel Chi-square tests and the Bonferroni correction was applied.

Synchronisation of ObjP in a group of four pigs was assessed by the number of pigs playing simultaneously with a toy on the ground. Transition rates for number of pigs playing with the toy were computed and differences between groups were estimated by Cochran-Mantel-Haenszel Chi-square test. Synchronisation of SocP and LocP within a group was quantified by comparing the observed and expected probability that two or more pigs simultaneously show SocP or LocP. The 95% confidence limits were computed.

## Supplementary information

Supplementary information

## Data Availability

The datasets gathered during the current study are available on request from the corresponding author.
