# Supplementary material for: Individual play patterns stimulated by a familiar object are group-driven
Source: Sci Rep. 2019 Apr 15;9:6092. doi: 10.1038/s41598-019-42382-9 (PMC6465404; doi:10.1038/s41598-019-42382-9)
Supplement: Supplementary file 1 — Supplementary information [file 41598_2019_42382_MOESM1_ESM.docx]

**Individual play patterns stimulated by a familiar object are group-driven**

Manja Zupan^1*^, Therese Rehn^1^, Daiana de Oliveira^1^, Špela Malovrh^2^, Linda Keeling^1^

^1^Swedish University of Agricultural Sciences, Department of Animal Environment and Health, P. O. Box 7068, 750 07 Uppsala, Sweden

^2^ University of Ljubljana, Biotechnical Faculty, Department of Animal Science, Groblje 3, 1230 Domžale, Slovenia

Supplementary Table S1: Descriptions of the terms used in the manuscript in alphabetical order.

| Name | Description |
| --- | --- |
| Development of play during test | Changes in play types on a group level during the 30 min test |
| Elements of play | Specific activities related to play; Table 1 describes the elements related to the different play types |
| Group dynamics | On a group level: influenced by individual play pattern and type of player |
| Groups of pigs | Replicates: 15 groups of four pigs |
| Play pattern | On an individual level: a description of a pig’s play behaviour based on the proportion of time spent (calculated on absolute frequencies of states) in different play types and on the variation in sequences of play  Statistical term: cluster (type A, type B, type C). |
| Play states | Statistical term: play types and their combinations |
| Sequences of play | A list of play elements with a particular order |
| State sequence | Statistical term: a list of state elements with a particular order |
| Synchronisation of play | Number of animals playing at the same time |
| Type of player | Initiator, joiner, mixed player;  Solitary, social, mixed player |

Supplementary Table S2: The allocation of time spent (calculated on absolute frequencies of states) on play types during 30 min in a home pen play test between different groups of four pigs. Groups were ordered according to the time performed on ObjP. ObjP-object play; LocSocP-locomotor and social play; NoP-activities not related to play. I-initiator; J-joiner; I/J-mixed player.

| Group | Type of player | | | Play types (%) | | |
| --- | --- | --- | --- | --- | --- | --- |
| I | | J | I/J | ObjP | LocSocP | NoP |
| 1 | 0 | 2 | 2 | 64.4 | 4.8 | 30.8 |
| 2 | 2 | 2 | 0 | 63.5 | 8.7 | 27.9 |
| 3 | 0 | 2 | 2 | 52.7 | 4.7 | 42.6 |
| 4 | 0 | 2 | 2 | 52.7 | 10.8 | 36.5 |
| 5 | 0 | 2 | 2 | 52.2 | 9.7 | 38.2 |
| 6 | 0 | 2 | 2 | 52.2 | 5.1 | 42.7 |
| 7 | 0 | 1 | 3 | 51.9 | 11.9 | 36.2 |
| 8 | 1 | 2 | 1 | 51.2 | 6.6 | 42.2 |
| 9 | 0 | 2 | 2 | 50.4 | 7.1 | 42.5 |
| 10 | 1 | 2 | 1 | 45.1 | 8.2 | 46.7 |
| 11 | 0 | 2 | 2 | 39.7 | 8.5 | 51.9 |
| 12 | 0 | 3 | 1 | 39.2 | 12.1 | 48.8 |
| 13 | 1 | 1 | 2 | 34.4 | 1.7 | 63.8 |
| 14 | 1 | 1 | 2 | 25.6 | 5.3 | 69.2 |
| 15 | 0 | 2 | 2 | 25.2 | 8.3 | 66.5 |

Supplementary Table S3: The significant effects of group of pigs and type of player (initiator, joiner or mixed type of player) on the sequence of play observed for 30 min in a home play pen test in 15 groups of four pigs. PseudoF and PseudoR^2^ are the two statistics in TraMineR for the analysis between groups of sequences. PseudoR^2^ explains the % of data variation.

| Effect | No. of levels | PseudoF | PseudoR^2^ | P-value |
| --- | --- | --- | --- | --- |
| Group | 15 | 1.52 | 32 | 0.0002 |
| Type of player | 3 | 1.72 | 5 | 0.0012 |
| Total |  | 1.55 | 37 | 0.0002 |

Around 70% of data variation could be explained by the full model.

Supplementary Table S4: The analysis of variance in the sequences for turbulence (variance of the durations of the successive states), complexity index (geometric mean between the normalized entropy and the length of the distinct successive states normalized by the length of the sequence) and longitudinal entropy (diversity of states that composes the sequence) by 15 groups of four pigs and type of player. I-initiator; J-joiner; I/J-mixed type of player.

| Effect |  | Turbulence | | Complexity index | | Longitudinal entropy | |
| --- | --- | --- | --- | --- | --- | --- | --- |
|  | df | F-value | P-value | F-value | P-value | F-value | P-value |
| Group | 14 | 4.49 | ≤0.0001 | 4.73 | ≤0.0001 | 2.73 | 0.006 |
| Type of player | 2 | 18.09 | ≤0.0001 | 19.16 | ≤0.0001 | 6.95 | 0.002 |
|  |  | Estimate | P-value | Estimate | P-value | Estimate | P-value |
| Type of player | J-I | 25.64 | 0.00003 | 0.12 | 0.00001 | 0.11 | 0.0058 |
|  | I/J-I | 13.59 | 0.031 | 0.07 | 0.01 | 0.08 | 0.08 |
|  | J-I/J | 12.054 | 0.001 | 0.05 | 0.002 | 0.036 | 0.20 |

Supplementary Table S5: Transition rate matrix of play types during 30 min in a home pen play test by type of player; ^a,b^Percentages in the same column for a particular behaviour lacking a common superscript letter differ by P˂0.05. NoP-activities not related to play; ObjP-object play; LocSocP-locomotor and social play. Values in bold present total transition rates of the same play types.

| Preceding behaviour | Type of player | Following behaviour (%) | | |
| --- | --- | --- | --- | --- |
|  |  | →NoP | →ObjP | →LocSocP |
| NoP→ | Initiator | 87.31^a^ | 9.14^a^ | 3.55^a^ |
|  | Joiner | 79.74^b^ | 13.99^b^ | 6.27^b^ |
| Total |  | **79.4** | 14.7 | 5.9 |
| ObjP→ | Initiator | 4.51^a^ | 93.08^a^ | 2.41^a^ |
|  | Joiner | 13.79^b^ | 80.76^b^ | 5.46^b^ |
| Total |  | 13.6 | **81.9** | 4.5 |
| LocSocP→ | Initiator | 31.52^a^ | 31.52^a^ | 36.96^a^ |
|  | Joiner | 40.16^a^ | 24.05^a^ | 35.78^a^ |
| Total |  | 39.9 | 24.1 | **36.0** |

Supplementary Table S6: Transition rate matrix of the number of individual pigs playing with a toy on the ground (ObjP) in 15 groups of four pigs during 30 min in a home pen play test presented as forward change. Diagonal values are presented in bold and show the high stability of group size in ObjP. Above the diagonal, the number of pigs involved in play increases and below the diagonal, the number of pigs decreases.

| Preceding no. of pigs | Following no. of pigs (%, n) | | | | |  |
| --- | --- | --- | --- | --- | --- | --- |
|  | →0 | →1 | →2 | →3 | →4 | Total |
| 0→ | **88.70 (613)** | 8.10 (56) | 2.32 (16) | 0.72 (5) | 0.14 (1) | 691 |
| 1→ | 4.23 (57) | **68.10 (918)** | 19.58 (264) | 6.23 (84) | 1.85 (25) | 1348 |
| 2→ | 0.73 (11) | 17.60 (264) | **50.87 (763)** | 22.93 (344) | 7.87 (118) | 1500 |
| 3→ | 0.17 (2) | 7.55 (89) | 30.11 (355) | **45.72 (539)** | 16.45 (194) | 1179 |
| 4→ | 0.00 (0) | 3.15 (21) | 16.04 (107) | 31.18 (208) | **49.63 (331)** | 669 |
| Total | 683 | 1348 | 1505 | 1180 | 669 | 5385 |
